# Intranasal infection of Rift Valley fever virus in the ferret model suggests multiple routes of neuroinvasion with consequential encephalitis and ophthalmitis

**DOI:** 10.1038/s41598-026-53431-5

**Published:** 2026-05-27

**Authors:** Audra-Lynne D. Schlachter, Karen L. Mansfield, Estela Gonzalez, Alejandro Núñez, Pedro J. Sánchez-Cordón, Fabian Z. X. Lean, Ignacio Garcia-Bocanegra, Alejandro Brun, Nicholas Johnson

**Affiliations:** 1 Animal and Plant Health Agency, Woodham Lane, New Haw, Addlestone, Surrey UK; 2 Department of Pathobiology and Population Sciences, Royal Veterinary College, North Mimms, Hertfordshire, UK; 3 Centro de Investigación en Sanidad Animal (CISA-INIA/CSIC), Valdeolmos, 28130 Madrid, Spain; 4 Grupo de Investigación en Sanidad Animal y Zoonosis (GISAZ), Departamento de Sanidad Animal, UIC Zoonosis y Enfermedades Emergentes ENZOEMUniversidad de Córdoba, 14014 Córdoba, Spain; 5 ISCIII de Enfermedades Infecciosas, CIBERINFEC, Instituto de Salud Carlos III, 28029 Madrid, Spain; 6 Faculty of Health and Medical Sciences, University of Surrey, Guildford, Surrey UK; 7 Department of Infectious Diseases and Public Health, Jockey Club College of Veterinary Medicine and Life Sciences, City University of Hong Kong, Kowloon, Hong Kong SAR China

**Keywords:** Rift Valley fever virus, Ferret, Intranasal, Encephalitis, Ophthalmitis, Anterior uveitis, Diseases, Microbiology, Neurology, Neuroscience

## Abstract

Rift Valley fever virus (RVFV) causes haemorrhagic fever, ophthalmitis, and in severe cases, encephalitis in humans. Despite the well-recognised neurotropism, the mechanisms of central nervous system (CNS) invasion remain poorly understood. We investigated RVFV ingress into the CNS and resulting neuropathogenesis following intranasal inoculation in ferrets (*Mustela putorius furo*) (*n* = 15). At 3 DPI, before RVFV dissemination to the CNS, mild multifocal suppurative rhinitis and multifocal interstitial pneumonia were detected in the nasal cavity and lungs respectively, supported by detection of viral antigen and genome. From 6 DPI, infected ferrets exhibited neurological signs; by 6 − 8 DPI, a mild-to-moderate non-suppurative meningoencephalitis and myelitis was observed, progressing rostrally to caudally, with ophthalmitis observed from 7 DPI and 100% mortality by 8 DPI. Mild-to-moderate hyperaemia of the meninges was identified macroscopically in all infected ferrets. An immune response to infection was unable to prevent neuropathogenesis and clinical disease. Although our results suggest that neuroinvasion occurs via the olfactory route through the nasal turbinates to cranial nerves, virus replication in the lungs and presence of viremia implies that haematogenous dissemination into the CNS as a secondary route is possible. This relevant experimental ferret model may further contribute to understanding the pathogenesis of human RVFV infection.

**Supplementary Information:**

The online version contains supplementary material available at https://doi.org/10.1038/s41598-026-53431-5.

## Introduction

Rift Valley fever virus (RVFV) is a mosquito-borne *Phlebovirus* within the class *Bunyaviricetes*, order *Hareavirales*, and family *Phenuiviridae*^1,2^. RVFV is the causative agent of Rift Valley fever (RVF) and was first reported in the Great Rift Valley of Kenya in 1931^3^. The virus has been detected in several countries across Africa and the Arabian Peninsula over recent decades, and subsequent outbreaks have affected the Indian Island of Mayotte following importation of cattle^4^, highlighting the transboundary potential of RVFV. There are currently no licensed human vaccines against RVFV, and although a range of veterinary vaccines are available, several disadvantages limit their use^5,6^.

RVFV transmission in both wild animals, such as rodents and African buffalo^7^, and domestic animals, including cattle, sheep, goats, and camels, is primarily through bites from infectious mosquitoes^8^. Infection can lead to high levels of morbidity and mortality during outbreaks, with abortion rates of up to 100% in some species due to transplacental foetal infection and placentitis^9,10^. While mosquito-borne transmission is important for epizootic outbreaks in humans and animals, humans may also acquire mucosal infection through direct contact with, and inhalation of, droplets arising from infected animal tissue and fluids, particularly during slaughter or butchery of infected carcases, or veterinary practices such as assisting an animal with parturition or handling aborted foetal material^11,12^.

Most human infections with RVFV are asymptomatic or result in a mild illness including fever, headache, and arthralgia. However, severe disease can manifest as haemorrhagic fever, encephalitis, ophthalmitis or hepatitis, and occurs in around 10% of human cases^13,14^. Among these, neurological disease is one of the most severe manifestations of infection with RVFV in humans, and can sometimes be fatal^14^, yet the pathogenesis of encephalitis is still poorly characterised. The study of encephalitis induced by RVFV has historically been limited to small laboratory animal models such as rodents that have produced variable disease outcomes^15,16^, or non-human primates, which recapitulate human disease more effectively but can be difficult to reproduce experimentally^17^. Given the occupational risks posed to veterinarians, farmers, and abattoir workers through inhalational exposure to RVFV contaminated bodily fluids, a better understanding of the neurologic and ophthalmic pathogeneses following respiratory exposure is needed.

The ferret (*Mustela putorius furo*) has previously been used to model human respiratory and neuropathogenic viruses, and experimental studies with influenza virus and SARS-CoV-2 have demonstrated neuroinvasion through the nasal cavity into the central nervous system (CNS), comparable to processes reported in humans^18^. Recently, Barbeau et al^19^. demonstrated that ferrets inoculated intranasally with a recombinant wild type virus derived from RVFV strain ZH501 developed neurological disease between 8- and 11-days post-infection (DPI), characterised by microscopic evidence of meningoencephalitis with concomitant high viral loads. However, key aspects of RVFV neuropathogenesis in this model remain to be addressed, including the mechanisms of neural and ocular invasion, the role of the mucosal-neural interface, and viral shedding dynamics within the nasal cavity, which may influence onward transmission. In this study, we investigate RVFV pathogenesis in ferrets following intranasal inoculation (mucosal infection), focusing on neural and ophthalmic pathogenesis, host immune responses and potential involvement of the host cell attachment factor low-density lipoprotein receptor-related protein 1 (LRP1), which has been shown to be associated with RVFV entry into cells^20^. This work will build on previous studies^19^ to further delineate the neuropathogenesis of RVFV in the ferret model as a proxy for RVFV-induced CNS disease in humans.

## Results

### Intranasal RVFV infection in ferrets resulted in rapidly progressive neurological disease

**Fig. 1:**
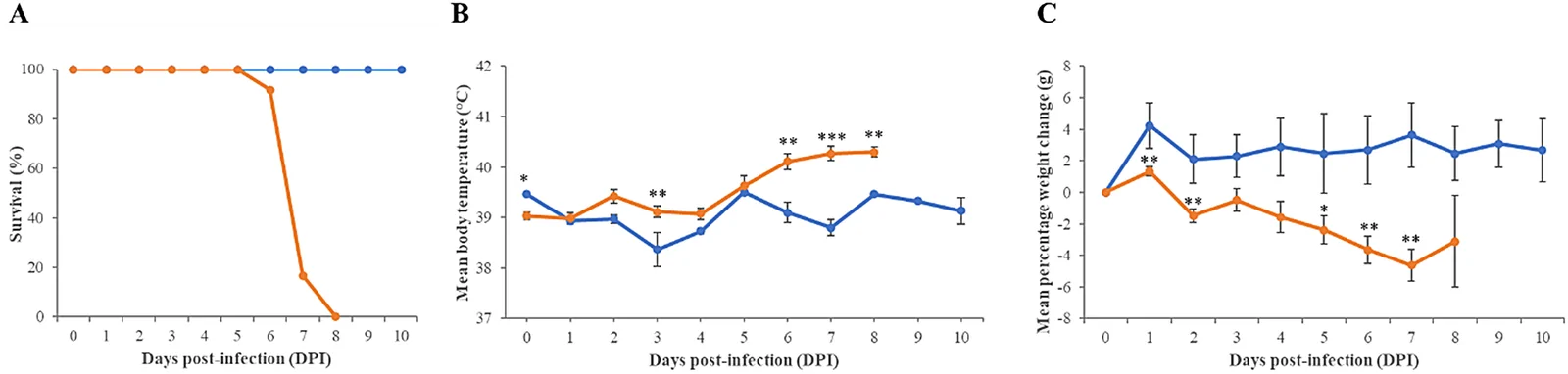
Clinical course in ferrets following intranasal infection with RVFV over the duration of the study, showing **A** survival (%), **B** mean body temperatures (°C), and **C** mean percentage change in bodyweight in comparison to weight at 0 DPI (g) and RVFV-infected ferrets denoted by an orange line, and uninfected control ferrets by a blue line. Statistically significant differences between mean body temperature for uninfected and RVFV-infected ferrets at each timepoint were assessed by unpaired t-test (two-tailed). Statistically significant differences in percentage weight change (in relation to baseline at 0 DPI) for uninfected ferrets and RVFV-infected ferrets at each timepoint (mean ± SEM) were assessed by unpaired t-test (two-tailed). Error bars denote SEM, where applicable; *p < 0.05, **p < 0.01, ***p < 0.001.

Intranasal RVFV infection resulted in subclinical infection between 0 − 3 DPI followed by rapid onset of clinical signs, with clinical endpoints (resulting in euthanasia) reached by 6 − 8 DPI (Table 1; Fig. 1A). A group of three ferrets were euthanised and subject to necropsy at 3 DPI, a planned subclinical endpoint. No clinical signs were apparent in any of these infected ferrets, apart from one animal that had developed pyrexia (defined as body temperature > 40.1 °C) from 2 DPI (Table 1; Fig. 1B). After this initial observation episodic or persistent pyrexia was subsequently detected in ten additional ferrets by 8 DPI. From 3 DPI onwards, statistically significant differences in body temperature were detected in RVFV infected ferrets when compared to the uninfected control group, at time-points of 3 DPI (*p* = 0.0082), 6 DPI (*p* = 0.0096), 7 DPI (*p* = 0.0001) and 8 DPI (*p* = 0.0023) (Table 1; Fig. 1B). Similarly, from 3 DPI, and during the clinical phase of infection, a steady decline in body weight (reflected as percentage change in comparison to values at 0 DPI) was observed in all remaining infected ferrets (*n* = 12) (Fig. 1C).

**Table 1:** Summary of clinical signs and clinical scores in ferrets inoculated intranasally with RVFV.

		RVFV-infected ferrets				Uninfected control ferrets
		Planned endpoint	Clinical endpoints			
		3 DPI ^a^ *(n* = 3)	6 DPI *(n* = 1)	7 DPI *(n* = 9)	8 DPI *(n* = 2)	10 DPI *(n* = 3)
Clinical signs	Pyrexia ^b^	1 (33.3)	0 (0)	8 (88.9)	2 (100)	0 (0)
	Dyspnoea	0 (0)	1 (100)	2 (22.2)	0 (0)	0 (0)
	Neurological signs	0 (0)	1 (100)	9 (100)	2 (100)	0 (0)
Clinical scores	Mean	0	10	9.7	9.5	0
	Range	N/A	N/A	3 − 15	9 − 10	N/A
	SEM	N/A	N/A	1.3	0.5	0

Data is presented as the number of RVFV-infected ferrets (percentage in parentheses) showing clinical signs at planned endpoint (3 DPI) and on reaching clinical endpoints (6 − 8 DPI), in comparison to uninfected control ferrets at 10 DPI. Clinical scores (mean and range) at each timepoint also shown. Total number of ferrets humanely killed at each timepoint denoted by *n*. ^a^Group of three ferrets humanely killed at planned timepoint of 3 DPI; ^b^ Body temperature > 40.1 °C. *N/A* not applicable, *SEM* Standard Error of the Mean.

RVFV infection led to weight loss over the duration of the study, where the percentage weight changes (in relation to 0 DPI baseline values) for RVFV-infected ferrets were significantly different when compared to values for uninfected control ferrets at time-points from 1 DPI to 7 DPI (*p* < 0.05 to *p* = 0.025) (Table 1; Fig. 1C). One RVFV-infected ferret achieved a 9.6% reduction in bodyweight by clinical endpoint (7 DPI), when compared to its baseline weight at 0 DPI. Dyspnoea was detected in three ferrets at 6 and 7 DPI (Table 1). Neurological signs suggestive of multifocal CNS disease were also seen from 6 DPI in all remaining infected ferrets (*n* = 12), including abnormal behaviour, reluctance to walk, limb weakness, reduced responsiveness, squinting, ataxia, and seizures.

In summary, intermittent pyrexia occurred in early infection, whereas consistent pyrexia, dyspnoea and neurological signs occurred concomitantly from 6 DPI in RVFV infected ferrets. The clinical picture deteriorated rapidly with increasing clinical scores from 6 DPI, and clinical endpoints were reached between 6 and 8 DPI. RVFV infection therefore resulted in 100% morbidity and subsequent mortality in infected ferrets (*n* = 12) by 8 DPI (Fig. 1A). Full details of scoring, clinical signs, clinical scores and gross post-mortem findings are shown in Supplementary Tables S1 and S3.

### Gross pathology changes in RVFV-infected ferrets were non-specific and limited to the brain, lymph nodes and lungs

Macroscopic evaluation performed during necropsy at each timepoint revealed that in all infected ferrets sampled (*n* = 15), the most striking finding was hyperaemia of the leptomeninges which was apparent from 3 DPI (Supplementary Fig. S1). Mild lymphadenomegaly affecting the submandibular and retropharyngeal lymph nodes was also noted in all infected ferrets from 3 DPI. Moderate diffuse pulmonary congestion was frequently present at all timepoints but was attributed to the method of euthanasia (intracardiac injection under terminal anaesthesia).

### RVFV infection in ferrets induced inflammatory changes and rapid virus dissemination from nasal mucosa and lungs to brain, peripheral nervous tissues, and eyes

**Fig. 2:**
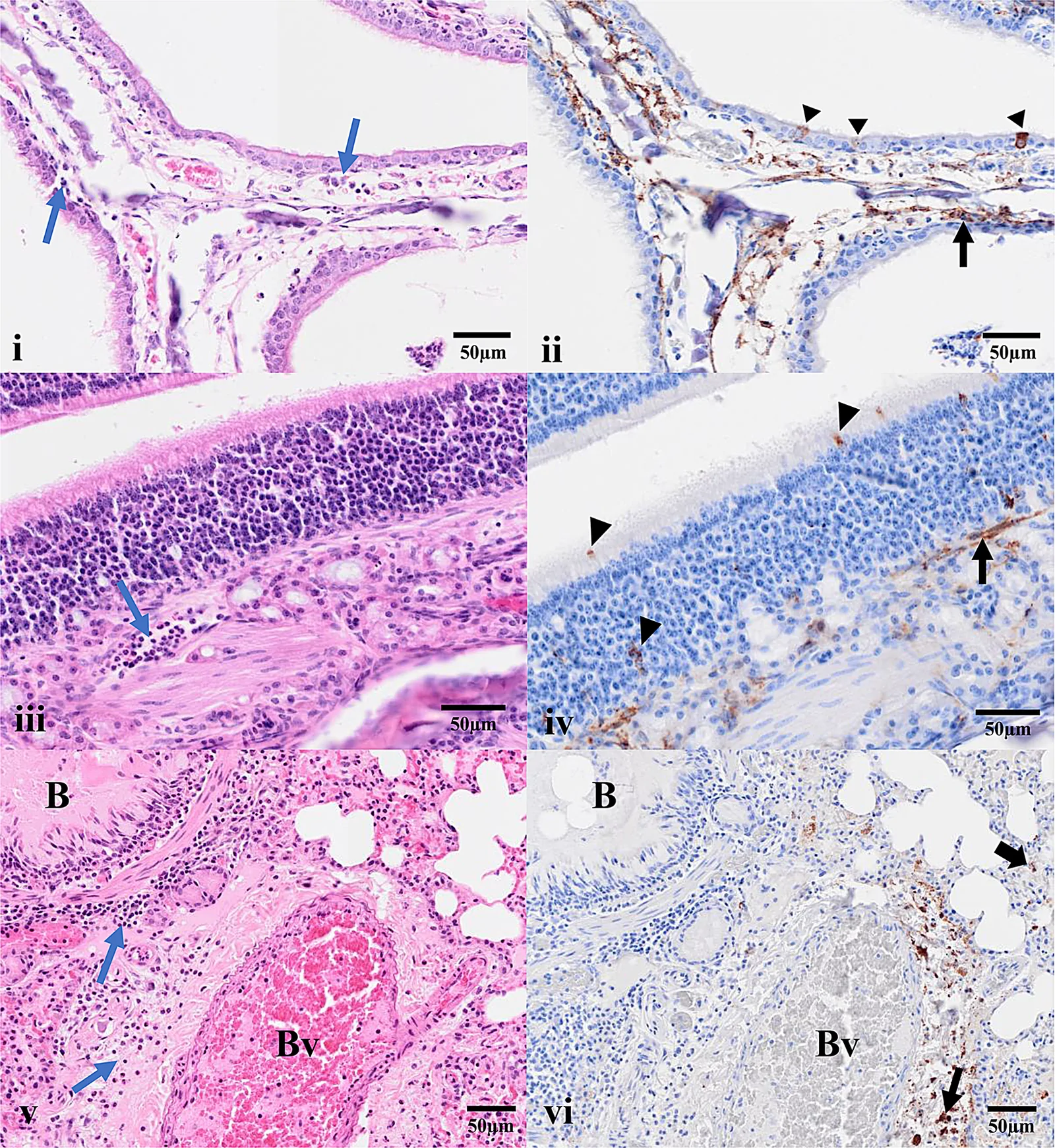
RVFV infection induces rhinitis and pneumonia in the ferret during the early stages of infection at 3 DPI. At 3 DPI in the respiratory nasal turbinates, (i) mild to moderate multifocal suppurative rhinitis () was observed, with ii) viral antigen detection (brown labelling) in ciliated epithelial cells and presumptive solitary chemosensory cells (), and associated nerve fascicles () seen on IHC. In the olfactory turbinates, mild multifocal suppurative rhinitis (iii, blue arrow) was evident with (iv) viral antigen detected in the mucosa within olfactory nerve fibres () and in the submucosa in nerve fascicles (). In the lungs, v) mild to moderate multifocal angiocentric pneumonia () was identified and (vi) immunolabelling detected in interstitial macrophages in the perivascular space () and occasionally in type 1 and 2 pneumocytes (). *Panels i*,* iii*,* v – sections stained with H&E. Panels ii*,* iv*,* vi - sections labelled with anti-RVFV antibody (brown labelling). Abbreviations: B - bronchiole*,* BV - blood vessel.*

Early in the course of infection at the 3 DPI planned endpoint, lesions were observed in all three ferrets in the nasal cavity and lungs, but no overt histopathological changes were detected in the CNS. Mild to moderate suppurative rhinitis was observed, in both the rostral (respiratory) and caudal (olfactory) (Fig. 2, i, iii respectively) nasal mucosa, characterised by neutrophilic infiltration of the submucosa, neutrophilic exocytosis, and exudation.

Viral antigen was detected in the nasal cavity in all three ferrets at 3 DPI, with greater abundance in the respiratory mucosa and submucosa than in the olfactory mucosa and submucosa, within nerve fascicles and sensory nerve fibres (Fig. 2, ii, iv). In the lungs, at 3 DPI (Fig. 2, v), the peribronchiolar and perivascular interstitium was multifocally mildly expanded by neutrophils and macrophages, and alveoli were variably filled with pale eosinophilic fluid (oedema) and fibrillar material. Adjacent bronchioles were also mildly infiltrated by neutrophils and macrophages, with infrequent epithelial disruption and neutrophilic exudation. Leucocytic margination across arteriolar walls and mild lymphocytic perivascular cuffing was evident. Infrequent immunolabelling was detected in the cytoplasm of macrophages within the pulmonary interstitium and intravascular spaces alongside histopathologic changes (Fig. 2, vi).

**Fig. 3:**
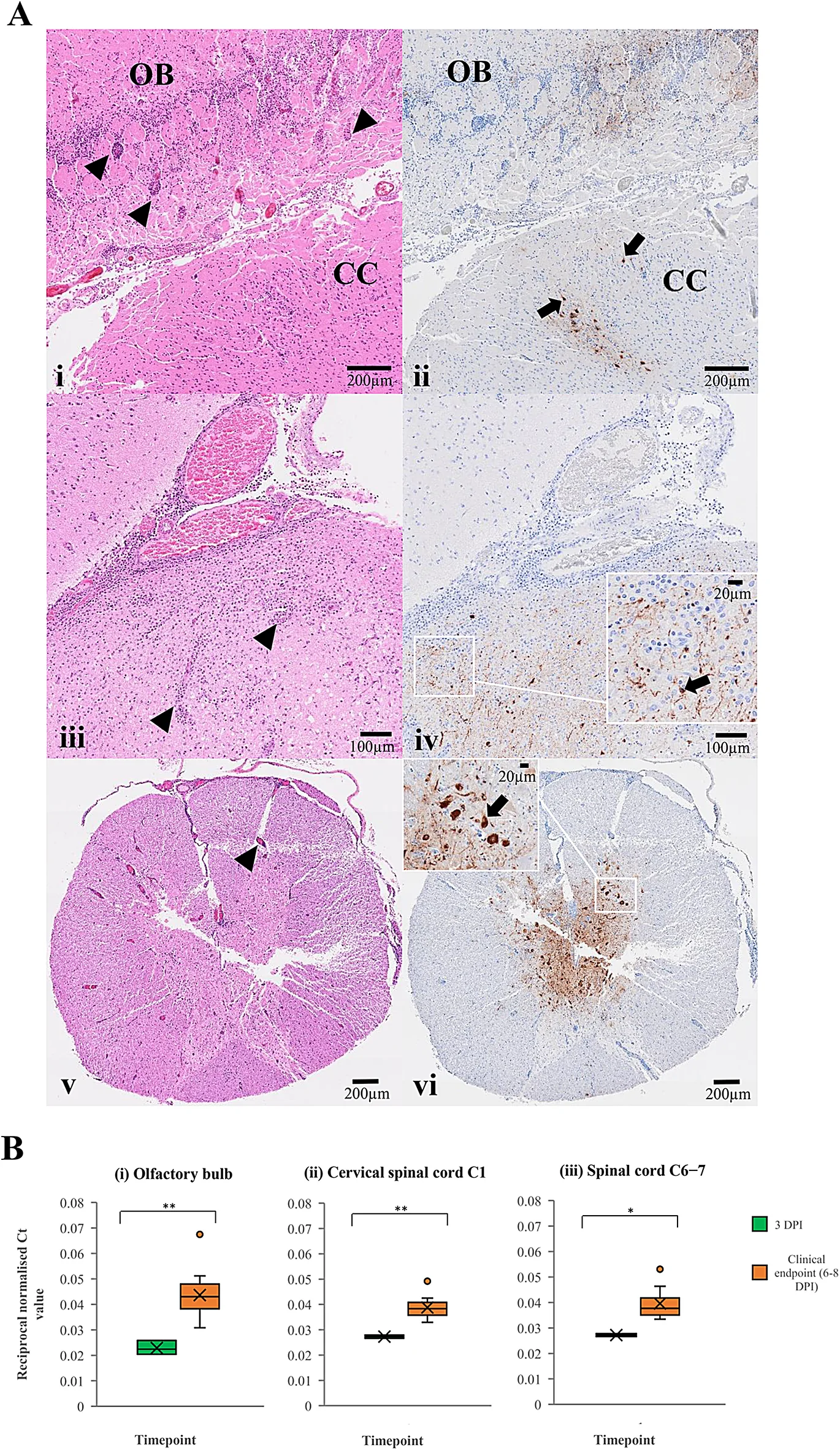
**A** Intranasal inoculation of ferrets with high dose RVFV leads to rapid and consistent infection in the CNS from 6 DPI. At 6 − 7 DPI, mild to marked multifocal lymphohistiocytic and neutrophilic meningoencephalomyelitis with moderate lymphohistiocytic perivascular cuffing (, i, iii and v) was observed, (i) in the olfactory bulb into the cerebral cortex (7 DPI), and throughout the remaining CNS as seen in (iii) the cerebral cortex (7 DPI) and (v) thoracic spinal cord (6 DPI). IHC of serial sections revealed abundant viral antigen (brown labelling) in neurons (, ii, insets iv and vi) and occasionally glial cells and the meninges in (ii) the olfactory bulb, (iv) cerebral cortex, and (vi) the grey matter of the spinal cord. *Panels ii*,* iv*,* vi - sections stained with a polyclonal anti-RVFV antibody (brown labelling). Panels i*,* iii*,* v – sections stained with H&E. Abbreviations: OB – olfactory bulb*,* CC – cerebral cortex.* **B** Detection of viral RNA increases in the CNS tissue as infection progresses. Reciprocal Ct values (normalised against β-actin) for RVFV detection in the olfactory bulb, cervical spinal cord C1 and spinal cord C6 − 7 at 3 DPI (subclinical [*n* = 3], green bars) and at 6 − 8 DPI (clinical endpoints [*n* = 12], orange bars). Statistical differences between subclinical and clinical timepoints were determined by unpaired t-test (two-tailed distribution). * *p* < 0.05, ** *p* < 0.01.

Only one ferret was euthanised at 6 DPI after reaching clinical endpoint, in which moderate multifocal rhinitis and mild multifocal interstitial pneumonia was also observed. In this ferret and all remaining eleven ferrets, IHC demonstrated a decline in viral antigen in the respiratory nasal mucosa and lungs between 6 and 8 DPI, but moderate immunolabelling in the olfactory nasal mucosa persisted until 8 DPI. Microscopic lesions in the CNS were first detected at 6 DPI, with moderate multifocal necrotizing meningoencephalitis seen in the olfactory bulb and rostral ventral olfactory tracts, and mild multifocal mixed inflammation observed in the cerebral cortex. From the level of the brainstem and caudally, moderate multifocal acute non suppurative encephalitis was identified in grey and white matter in the pons, cerebellum and in the hindbrain, and was present at all levels of the spinal cord (Fig. 3 A, v), predominantly in the grey matter.

From 7 DPI, encephalitis became more extensive (Fig. 3 A i, iii), with moderate to severe and prominent neuronal and glial cell death, marked lymphohistiocytic infiltration and mild to moderate lymphohistiocytic perivascular cuffing observed throughout the brain in the remaining eleven ferrets.

Mild to moderate viral antigen immunolabelling was observed in neurons and axonal processes and infrequently in glial cells in the olfactory bulb and cerebrum (Fig. 3A, ii, iv and iv inset), and to a lesser extent in the cerebellum, brainstem, and spinal cord (Fig. 3A, vi). The meninges were diffusely infiltrated with moderate numbers of lymphocytes and macrophages in most cerebral cortex sections examined (Fig. 3 A, iii, iv and iv inset), with infrequent viral antigen visible in leucocytes and in cells within the meninges, colocalised with histopathological changes. The increasing development of pathological changes in the CNS during the later stages of infection coincided with increasing detection of RVFV genome (Fig. 3B). Previous studies have shown a strong correlation between Ct values derived by RT-PCR and the titre of infectious RVFV^21,22^. Reciprocal normalised Ct values (1/Ct) were significantly increased (representative of lower actual normalised Ct values) once clinical signs were observed, between 6 − 8 DPI (*n* = 12) in (i) the olfactory bulb (*p* = 0.0034), (ii) cervical spinal cord (*p* = 0.0030) and (iii) C6 − 7 spinal cord (*p* = 0.0116), when compared to the earlier subclinical timepoint (3 DPI, *n* = 3). This suggests that RVFV disseminated into the olfactory bulb and spinal cord with increased viral replication as clinical course progressed. Although reciprocal Ct values are shown here for graphical representation, for the olfactory bulb, cervical spinal cord C1 and spinal cord C6 − 7, actual mean normalised Ct values were 44.1, 36.7 and 36.7 respectively at 3 DPI (SEM 3.0, 0.9 and 0.8), compared to 23.9, 26.1 and 25.7 respectively at 6 − 8 DPI (SEM 1.4, 0.7 and 0.9).

**Fig. 4:**
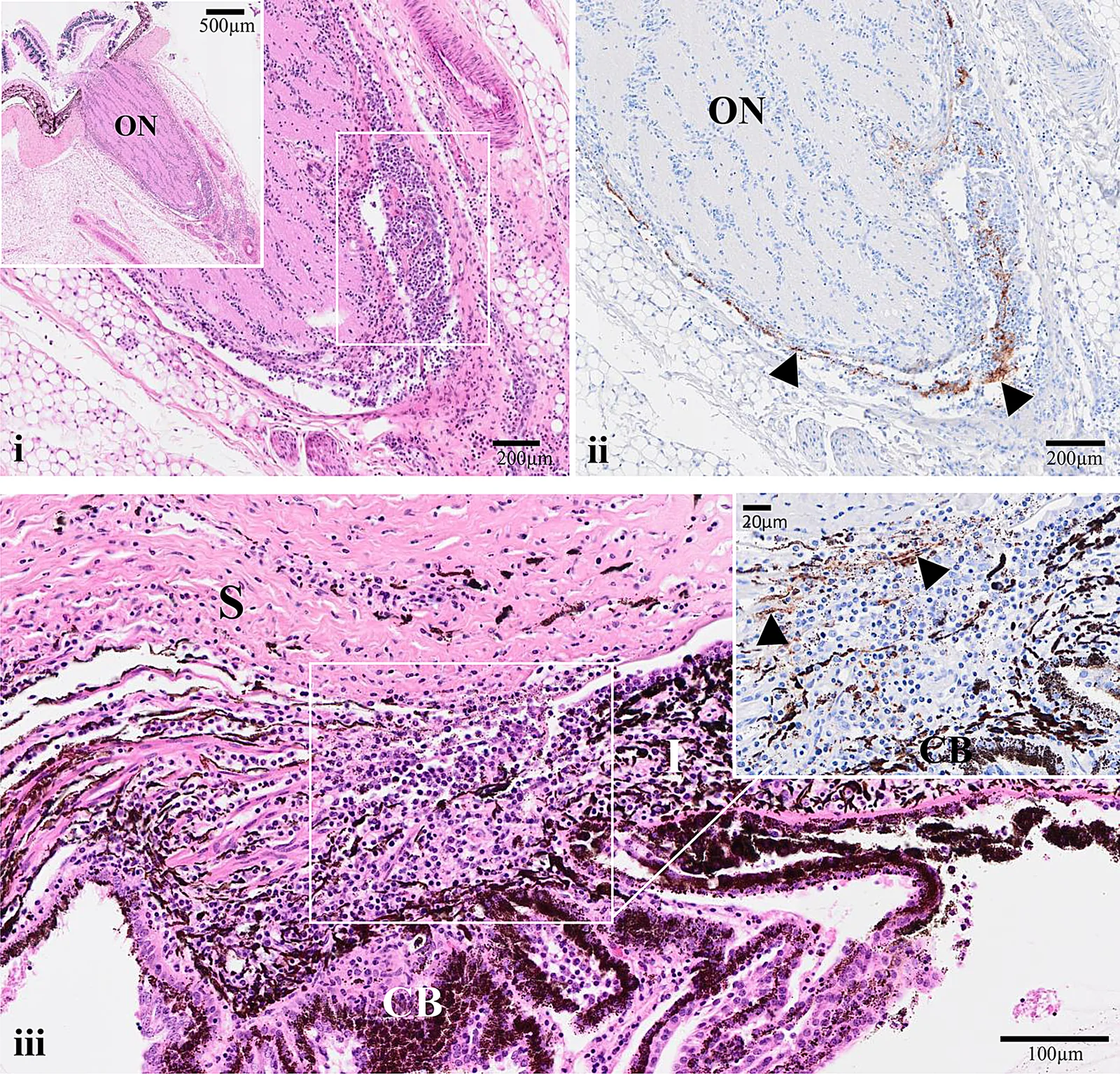
Iridocyclitis and optic neuritis associated with RVFV infection at 7 − 8 DPI. Ocular pathology following RVFV infection was characterised by (i) optic neuritis with perineural meningitis (white box) of the optic nerve (7 DPI) and (iii) mild to moderate non suppurative anterior uveitis (8 DPI, white box). Colocalised viral antigen was identified () in (ii) the meninges and (iii, inset) at the junction of the iris and ciliary body. *Panels i and iii stained with H&E*,* panel ii and iii inset strained with polyclonal anti-RVFV antibody (brown labelling). ON *optic nerve, *CB* ciliary body, *S* sclera, *I* iris.

In addition to CNS lesions, ocular pathology was identified in four ferrets at 7 and 8 DPI (Fig. 4). Mild to moderate lymphocytic peri-optic neuritis (Fig. 4i), colocalised with viral antigens (Fig. 4ii) in the arachnoid and dural membranes was detected at 7 DPI in three ferrets, with concomitant minimal to mild optic neuritis in two of these three ferrets. Moderate lymphocytic iridocyclitis (anterior uveitis, (Fig. 4iii), with co-localised viral antigen (Fig. 4iii inset), was observed in one ferret at 8 DPI.

No other significant lesions were observed in any of the remaining organs examined, although rare immunolabelling was detected in antigen presenting cells within germinal centres of lymphoid follicles in the submandibular lymph nodes (9/15 ferrets) at all time points, retropharyngeal (5/15 ferrets) lymph nodes at 3 DPI, and the spleen (2/15 ferrets) at 8 DPI.

### Correlative histopathology and virus immunohistochemistry demonstrated direct association between inflammatory changes in RVFV-infected ferrets and detection of RVFV antigen

**Fig. 5:**
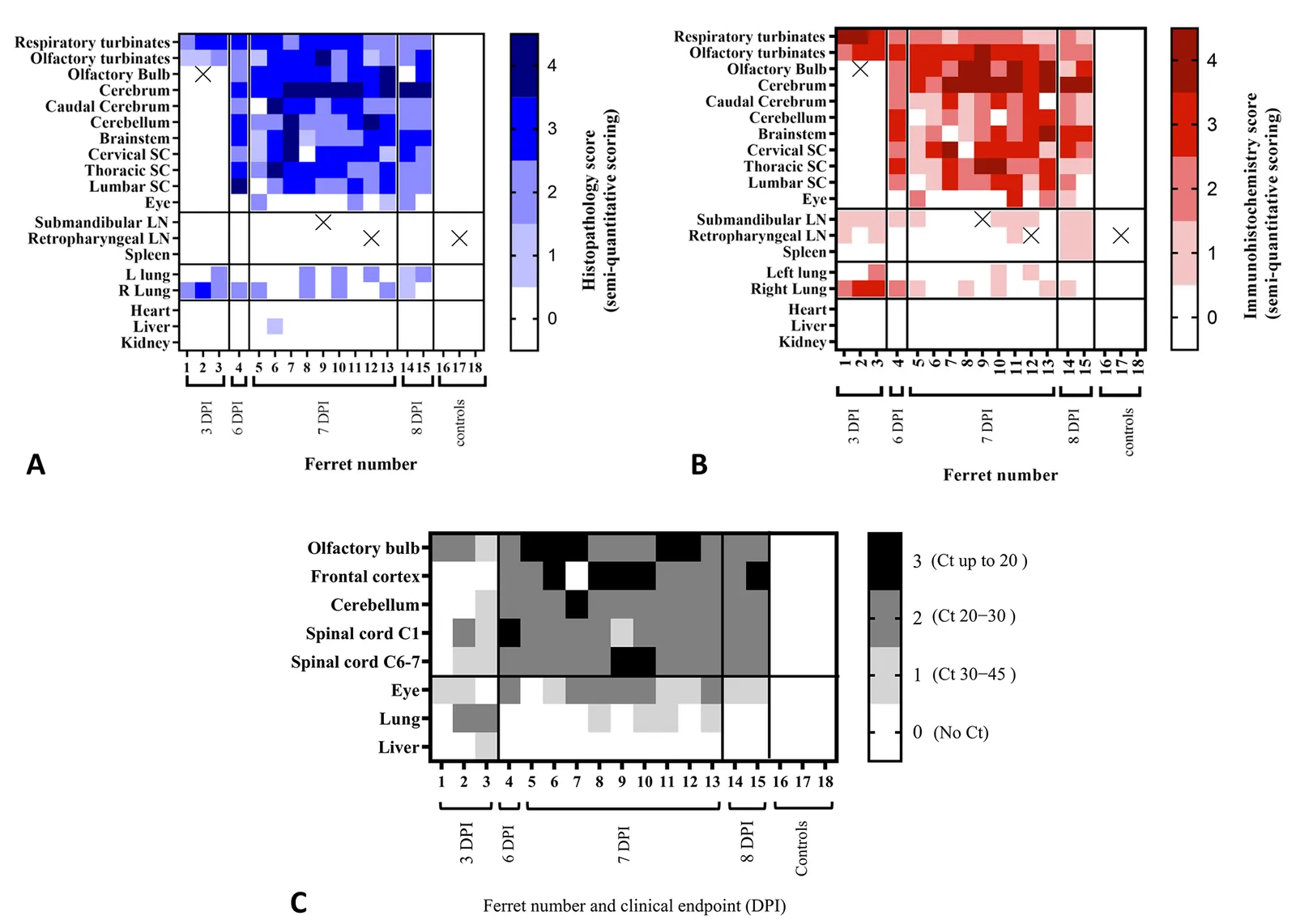
Semi-quantitative scoring of histopathological changes (**A**) and immunohistochemical staining for RVFV antigen (**B**). The distribution of inflammation and viral antigen in tissues is shown above, using a semiquantitative score from 0 − 4, where labelling is categorised as 0 (none), 1 (sparse), 2 (mild), 3 (moderate), and 4 (abundant). A cross indicates the tissue was not available for evaluation. Heat map representing the detection of viral genome by RT-PCR in ferret tissues (**C**). Visualisation based on Ct values less than 20 (3), Ct of 20 − 30 (2), Ct of 30 − 45 (1), or No Ct (0), for the subclinical endpoint (3 DPI, *n* = 3), clinical endpoints (6 − 8 DPI, *n* = 12) and uninfected controls (*n* = 3). There was no detection of viral RNA in uninfected ferrets (*n* = 3). *SC* spinal cord, *LN* lymph node.

Semi-quantitative scoring demonstrated that histopathology scores closely aligned with results from IHC antigen labelling in most tissues assessed, where inflammation was associated with the presence of viral antigen (Fig. 5A). The exceptions were the lymph nodes and spleen, where viral antigen was rarely detected in macrophages and there was no associated inflammation or lymphoid depletion.

IHC scoring (Fig. 5B) notably highlights abundant viral antigen detected in the respiratory mucosa during the early stages of infection, and the presence and in some cases, persistence, of antigen in the lungs from the subclinical incubation period (up to 3 DPI) to study end. It also demonstrates onset and progression of infection throughout the CNS and involvement of ocular structures from 7 DPI.

### RVFV establishes early neuroinvasion and disseminates through multiple routes

RVFV-specific RT-qPCR conducted on post-mortem tissues supported the histopathologic findings and provided further evidence that virus dissemination in the CNS occurred through multiple routes (Fig. 5C). At 3 DPI, although RVFV-infected ferrets were subclinical, RVFV RNA was already present in the lung (2/3 ferrets), olfactory bulb (3/3 ferrets), eye (2/3 ferrets), cerebellum (1/3 ferrets), and spinal cord (2/3 ferrets), but was not detected in the frontal cortex. However, at 6 − 8 DPI, RVFV RNA was observed throughout the olfactory bulb, cerebral cortex, cerebellum, spinal cord (C1, C6 − C7), eye, and the lung. Figure 5A-C clearly demonstrate the increasing RVFV invasion of the CNS in infected ferrets over the course of the study, accompanied by dissemination of virus to the eye and lung, but with minimal evidence of hepatic disease.

### Rift Valley fever virus was detected within the oral cavity and persisted in some animals

Molecular analysis of swabs demonstrated the presence of viral RNA in both oral and nasal swabs taken from all three ferrets humanely killed at 3 DPI (Supplementary Table S4). By clinical endpoints at 6 − 8 DPI, viral RNA was detectable in oral, nasal, and rectal swabs. Oral swabs tested at 3 DPI (subclinical, *n* = 3/3) showed significantly higher (*p* = 0.020) reciprocal normalised Ct values (mean = 0.033, SEM = 0.0002), representative of lower actual normalised Ct values (mean = 30.4, SEM = 0.19), when compared to the clinical endpoints at 6 − 8 DPI (*n* = 5/12) where the mean reciprocal normalised Ct value was 0.027 (SEM = 0.0013), representative of the actual mean normalised Ct value of 36.9 (SEM = 1.72) (Supplementary Fig. S2). This potentially suggests a reduction in virus shedding via the oral route over the course of infection. Viral RNA was also present in the nasal swabs analysed at both stages of infection (subclinical and clinical endpoints), although no significant difference was observed between reciprocal normalised Ct values obtained (*p* = 0.0730). During subclinical infection at 3 DPI the mean reciprocal normalised Ct value was 0.027 (SEM = 0.0004), equivalent to an actual mean normalised Ct value of 36.7 (SEM = 0.6), and at clinical endpoints (6 − 8 DPI) the mean reciprocal normalised Ct value was 0.030 (SEM = 0.0007), equivalent to an actual mean normalised Ct value of 33.5 (SEM = 0.7). This suggests that virus shedding by the nasal route remained consistent during the later stages of infection, with detectable viral RNA in 11/12 nasal swabs tested.

While the detection of viral RNA in oral and nasal swabs may be artefactual from intranasal inoculation, actual Ct values of 36.4 − 39.2 were also detected from rectal swabs obtained at clinical endpoint (6 − 8 DPI) in 3/12 ferrets (Supplementary Tables S4, S5). Although these molecular data suggest multiple routes for virus shedding, further analysis to isolate infectious virus from swabs would be required to provide absolute confirmation.

### RVFV infection in ferrets provided evidence for virus dissemination through viraemia

Viraemia was detectable at clinical endpoint (6 − 8 DPI), where infectious virus was isolated from three out of 12 infected ferrets by plaque assay, confirming low-level viraemia (range of titres 6.7–7.2 × 10^2^ PFU/ml). During the subclinical incubation period (3 DPI), and in uninfected control animals, infectious virus was not detectable in serum by plaque assay.

### RVFV infection in ferrets induced transcriptional up-regulation of host innate immune markers in the olfactory bulb and cerebellum and production of neutralising antibody

**Fig. 6:**
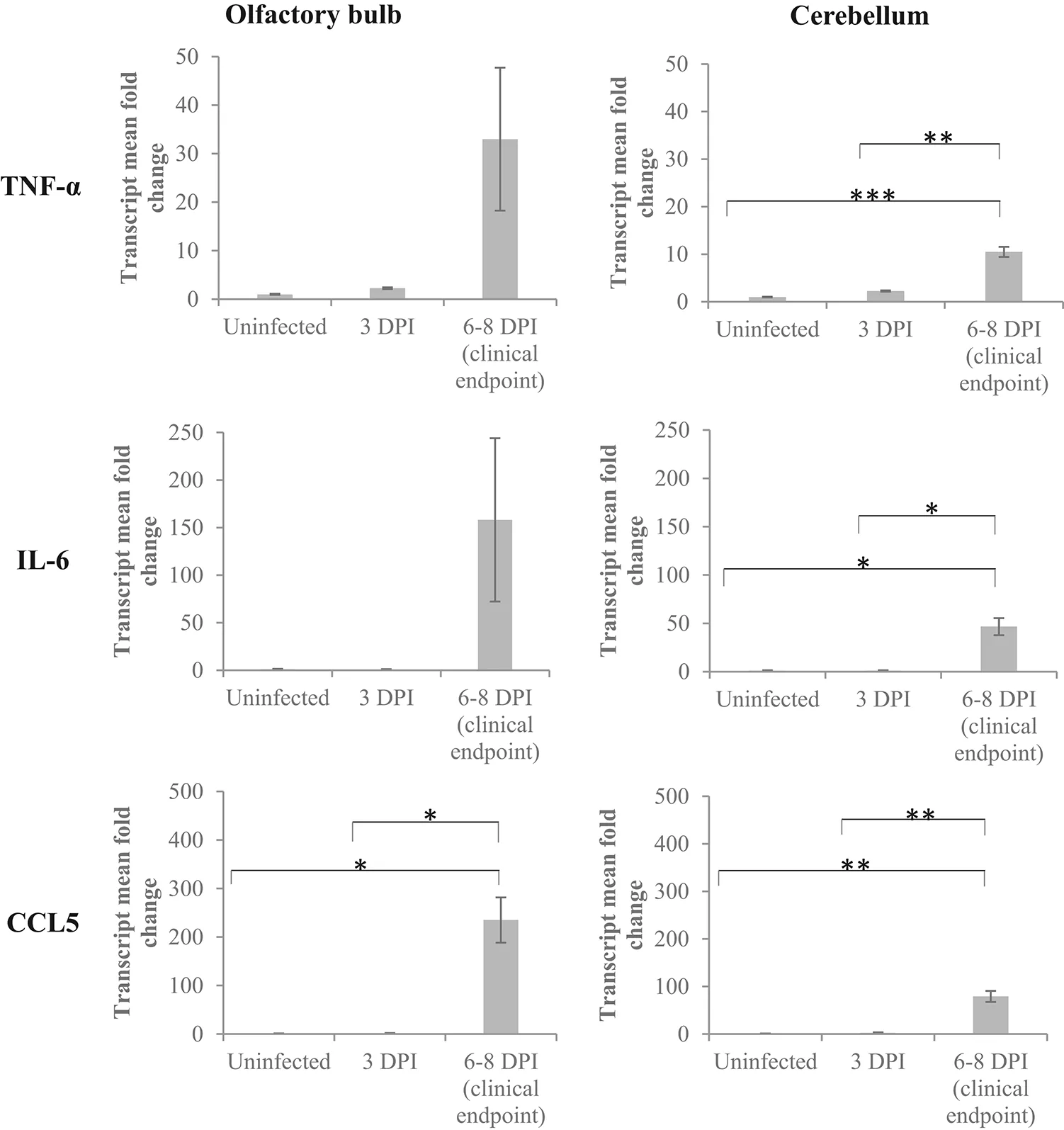
Pro-inflammatory innate immune transcripts (TNF-α, IL-6 and CCL5) in the olfactory bulb and cerebellum, following intranasal inoculation of ferrets with RVFV. Transcript mean fold change in comparison to the uninfected control group (*n* = 3) demonstrates significant transcriptional up-regulation in infected groups by clinical endpoint (6 − 8 DPI, *n* = 12). Statistical comparisons of mean fold-changes for preclinical and clinical endpoints (compared to the uninfected control group) were assessed using one-way ANOVA with Tukey’s comparison of means. Error bars denote SEM. * *p* < 0.05, ** *p* < 0.01.

Due to the predominance of neurological disease in RVFV-infected ferrets, the transcriptional response for key innate immune response mediators in selected CNS tissues was assessed. Fold change increases of host gene transcripts were investigated in the olfactory bulb and cerebellum for TNF-α, a pro-inflammatory cytokine associated with endothelial cell gene expression and neutrophil activation, IL-6, a pro-inflammatory cytokine produced by macrophages in response to infection, and CCL5, which plays an important role in the chemotaxis of lymphocytes, monocytes and eosinophils during viral infection. These analyses suggest low-level up-regulation of the pro-inflammatory cytokine response in the CNS during the early subclinical stage of infection (up to 3 DPI, *n* = 3), expressed as transcript mean-fold change (in comparison to uninfected control), which increased further as the course of infection progressed to clinical endpoint between 6 − 8 DPI (*n* = 12) (Fig. 6). For all host transcripts assessed, mean fold-change in comparison to the uninfected controls in both the olfactory bulb and cerebellum was minimal at 3 DPI, although TNF-α transcripts showed a 2.3-fold increase (not statistically significant) in both CNS tissues assessed (SEM of 0.2 and 0.1, respectively). This occurred prior to the development of clinical signs (Table 1) and coincided with detection of viral genome in the olfactory bulb in 3/3 ferrets at 3 DPI and the cerebellum in 1/3 ferrets at this timepoint (Fig. 5C). At this early timepoint, similar low-level non-significant fold-change increases were seen in both the olfactory bulb and cerebellum for CCL5 transcripts (1.9-fold [SEM 0.3] and 2.6-fold [SEM 1.0] respectively), although there was no change in IL-6 transcripts detected. However, by clinical endpoint (6 − 8 DPI), transcript mean fold changes for TNF-α and IL-6 in the cerebellum were significantly up-regulated in comparison to uninfected controls, with 10.5-fold (SEM 1.1, *p* = 0.0008) and 46.7-fold (SEM 9.3, *p* = 0.0424) increases, respectively. The increases in mean fold change at 6–8 DPI compared to 3 DPI (subclinical) were also significant for both TNF-α (*p* = 0.0026) and IL-6 (*p* = 0.0428), suggesting elevated levels of gene upregulation after 3 DPI, coinciding with clinical signs. In the olfactory bulb at 6 − 8 DPI, the mean transcript fold-change for IL-6 yielded a 158.1-fold (SEM 85.9) increase when compared to the uninfected control group, although this was not statistically significant (*p* = 0.5557).

Similarly, the 33-fold increase in mean TNF-α transcript fold-change in the olfactory bulb was not significant at 6 − 8 DPI (*p* = 0.5082). In comparison, analysis of CCL5 transcripts revealed significant transcript fold-change increases at clinical endpoint (6 − 8 DPI) in both the olfactory bulb (234.9-fold [SEM 46.6], *p* = 0.0374) and cerebellum (79.1-fold [SEM 11.4], *p* = 0.0073), indicative of the host antiviral response to facilitate leukocyte recruitment to sites of infection in the CNS as observed histologically (Fig. 3 A, i, iii and v). Again, upregulation of these genes occurred after 3 DPI, and were therefore also significant at 6 − 8 DPI when compared to 3 DPI for olfactory bulb (*p* = 0.0381) and cerebellum (*p* = 0.0084).

An adaptive humoral response to infection was also evident with the detection of a neutralising antibody response in most infected ferrets through RVFV-specific PRNT; two ferrets that were euthanised at 3 DPI (subclinical) did not mount a detectable antibody response. Neutralising antibodies against RVFV were therefore detected in 1/3 ferrets from the group sampled at 3 DPI (PRNT_90_ titre 1:20), and at 6 − 8 DPI in all remaining infected ferrets at clinical endpoint (PRNT_90_ titres of 1:10 to 1:80) (Supplementary Table S6). All pre-challenge bleeds (day 0) were negative by PRNT.

### RVFV infection in ferrets induced changes to the LRP1 transcript profile over the course of infection

**Fig. 7:**
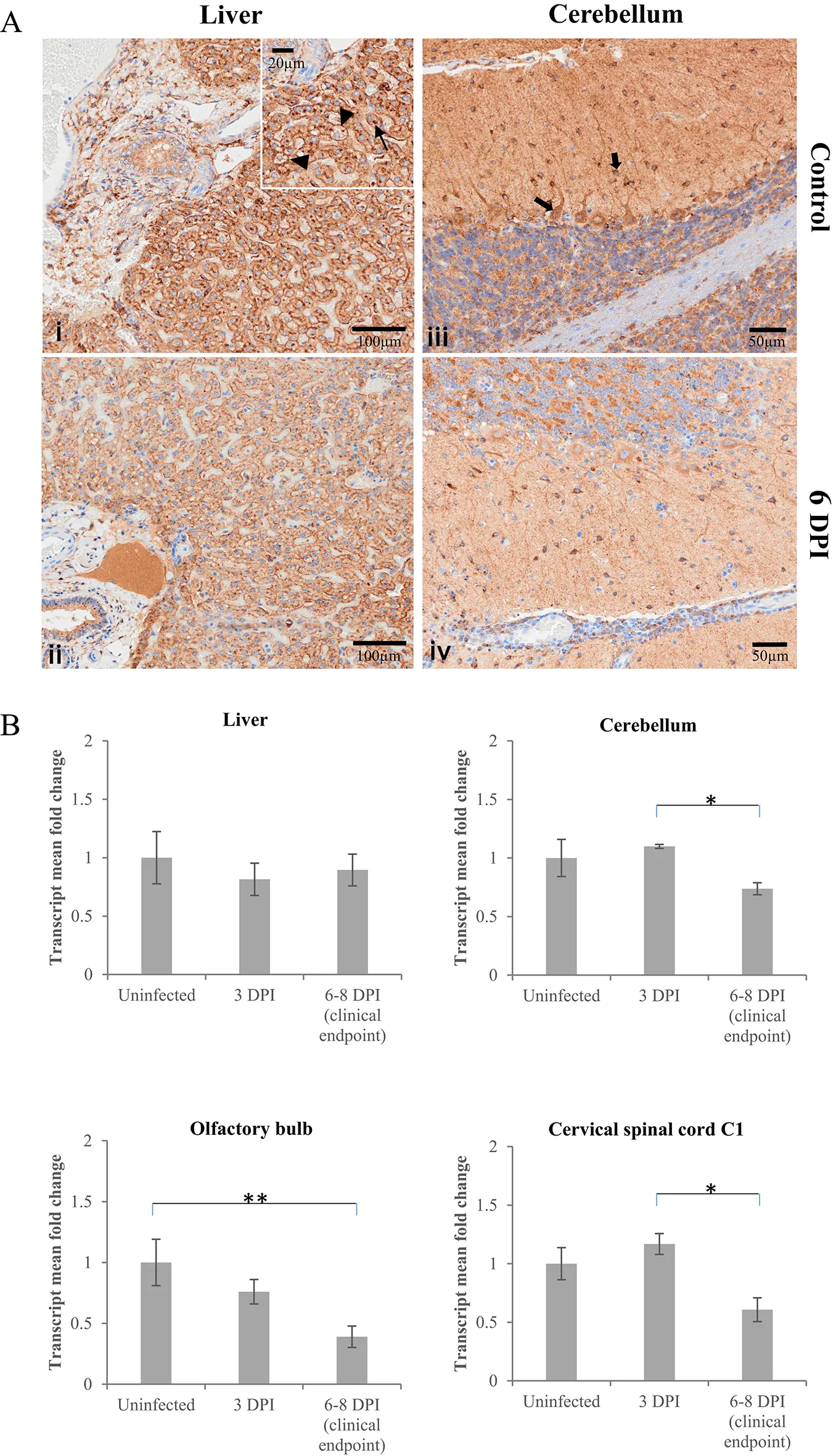
**A** Distribution of LRP1 (brown pigment) in the cytoplasm and plasma membrane of hepatocytes (liver, left column) and neurons (cerebellum, right column) in uninfected control ferrets and infected ferrets at 6 DPI. Diffuse positive labelling is present throughout the cytoplasm, with intense staining of the plasma membrane () and the perinuclear cytoplasm () of hepatocytes in the liver (i, ii, x20, inset x25). In the cerebellum (iii, iv x20), diffuse labelling of the neutrophil is observed and is concentrated in neuronal cell bodies (short black arrows). No observable difference noted between uninfected and infected ferrets. **B** Host gene transcripts for LRP1 in the liver, olfactory bulb, cerebellum, and cervical spinal cord C1, following intranasal inoculation of ferrets with RVFV. Transcript mean fold change in comparison to the uninfected control group (*n* = 3) demonstrates significant transcriptional down-regulation in infected groups by clinical endpoint (6 − 8 DPI, *n* = 12). Statistical comparisons of mean fold-changes for preclinical and clinical endpoints (compared to the uninfected control group) were assessed using one-way ANOVA with Tukey’s comparison of means. Error bars denote SEM. * *p* < 0.05, ** *p* < 0.01.

LRP1 immunohistochemistry was used to highlight the presence of the RVFV host cell attachment receptor in the cytoplasm and cell membrane of the liver and brain of control ferrets, those infected at 3 DPI (subclinical, *n* = 3) and 6 − 8 DPI (clinical endpoints, *n* = 12) (Fig. 7A). In control ferrets, LRP1 labelling was ubiquitously distributed as fine stippling in the cytoplasm of hepatocytes, bile duct epithelium, and endothelium in the liver, with signal concentrated in the cell membrane and often the perinuclear cytoplasm of hepatocytes. In the brain, LRP1 was identified within the cytoplasm of neuronal cell bodies and diffusely throughout the neuropil of the grey matter, and infrequently in axonal processes in the white matter. No discernible difference in immunolabelling was observed between control and infected ferrets in the liver or the cerebellum.

In contrast, LRP1 transcripts, reflected as transcript mean fold change in comparison to the uninfected control, demonstrated significant downregulation at clinical endpoint (6 − 8 DPI) in the cerebellum and spinal cord (Fig. 7B). These included the olfactory bulb, where transcript mean fold change was significantly downregulated at clinical endpoint in comparison to the uninfected control (*p* = 0.0070). Significant transcript downregulation was also observed in the cerebellum and cervical spinal cord at clinical endpoint, when compared to subclinical animals at 3 DPI (*p* = 0.0194 and *p* = 0.0357, respectively). In comparison, the mean fold change for LRP1 transcripts in the liver did not change significantly when compared to the uninfected control, at either 3 DPI (*p* = 0.8623) or clinical endpoint (*p* = 0.9255).

## Discussion

Current understanding of the neuropathogenesis of RVFV in humans is incomplete, as the disease is typically self-limiting, with neurological and ophthalmologic complications seen only in a small number of severe cases, and autopsy materials rarely available for investigation^14,23–25^. To deepen knowledge about the pathogenesis of RVFV infection and reinforce the utility of the ferret model to mimic RVFV-induced CNS disease in humans, we infected ferrets by intranasal inoculation to mimic mucosal infection through inhalation. While no clinical signs were observed during the initial stage of infection, ferrets subsequently went on to develop pyrexia, dyspnoea, and neurological disease, where the immune response was insufficient to control systemic virus dissemination and neuroinvasion. The intranasal route of inoculation therefore provided a biologically meaningful experimental model to simulate the risk of respiratory mucosal route of infection in humans following exposure to biological fluids of infected animals^26–28^. These data highlight the value of the ferret model to mimic RVFV-induced CNS disease in humans, in comparison to rodent models where diverse pathological changes occur depending upon the rodent species^29^.

Our findings suggest that, following intranasal inoculation, RVFV followed predominantly neuronal pathways into the CNS. IHC and RT-PCR demonstrated abundant viral antigen and RNA in the respiratory and olfactory nasal mucosa, and in olfactory nerve fibres in the submucosa, and following likely subsequent retrograde transport through the cribriform plate, abundant labelling in the olfactory bulb with onward dissemination to the CNS. Immunolabelling was visible in olfactory receptor neurons and submucosal nerve fibres as well as in a perineural location. Although not investigated further in this study, this perineural labelling could speculatively represent olfactory ensheathing cells (OEC). OEC form channels through which olfactory nerve fibres cross the submucosa through the cribriform plate and connect with the cerebrospinal fluid (CSF). These conduits have been proposed as a direct access route into the CNS for viral particles of diameter 100 nm or less, such as influenza virus, West Nile virus and bunyaviruses^30^. Alternatively, direct infection of OEC and subsequent viral release may occur^18^. This could account for the early histological appearance of meningitis in our study, where the virus reached the CSF around the olfactory bulb early in infection, as suggested previously following intranasal inoculation of highly pathogenic avian influenza A virus (HPAI) H5N1 in ferrets^31^.

There is evidence to suggest that virus passage potentially took place through peripheral cranial nerve V, as at 6 DPI, abundant viral antigen was seen in the nasal mucosa, olfactory bulb, olfactory tracts and meninges, and in the region of the trigeminal nerve nucleus in the pons with extension into the white matter tracts of the cerebellum, but infrequently observed in the cerebral cortex. Furthermore, the trigeminal nerve innervates the meninges, which may have contributed to the severity of the meningeal inflammation observed. Viral antigen was also found in the medulla oblongata and in the cervical, thoracic, and lumbar spinal cord at 6 DPI, indicating rapid neuroinvasion. Although these pathways were not mechanistically verified, neuroinvasion appears the more likely route of infection, as no viremia was observed during the preclinical window. Similar observations were described following intranasal inoculation of ferrets with HPAI H5N1, where infection efficiently reached the brain via olfactory and peripheral nerve pathways and spread by rapid anterograde dissemination to the rest of the CNS^32–34^.

While the neural route of CNS invasion is the most probable in this animal model, haematogenous transport of RVFV to the CNS following viraemia cannot be completely ruled out. A recent study using an in vitro model of the human blood-brain barrier (BBB) has highlighted the ability of RVFV to cross the BBB^35^. While no evidence of endothelial injury or endothelial labelling was seen in the brain in our study, occasional viral antigen positive macrophages in lymph nodes and in the lungs may suggest trafficking of infected monocytes and macrophages that may facilitate the haematogenous dissemination of RVFV into the brain.

Ocular disease is a common complication of RVF in humans^24^, ranging from retro-orbital pain, and blurred vision or even vision loss, secondary to uveitis, retinal haemorrhage, oedema and inflammation, and optic neuritis. In the ferret model, RVFV genome was detected in the eye as early as 3 DPI, and in almost all ferrets by 8 DPI, and a subset of four ferrets also demonstrated viral antigen associated uveitis and optic neuritis at 7 − 8 DPI. Unlike in the rat model in which posterior uveitis predominated^24^, only anterior uveitis was detected in the ferret model. In the absence of labelling in the conjunctiva, cornea or lacrimal glands, the neuroanatomical proximity of cranial nerves I (olfactory), II (optic), and III (oculomotor) may facilitate secondary anterograde spread into the eye, potentially accounting for development of optic neuritis and anterior uveitis. Outside of the nervous system, virus antigen was detected in the lungs, localised to the alveolar septa, macrophages, and adjacent vessel walls, most prominently at 3 and 6 DPI. This reflects virus dissemination to the pulmonary interstitium following infection of the nasal cavity, local multiplication, and establishment of viraemia. However, following direct intranasal inoculation of virus, and depending on several factors, particles may also travel deeper into the lower respiratory tract, and induce local infection and inflammation^36^.

In this study, despite the viraemia seen in the later stages of infection, the clinical course tended towards neurological disease, and no hepatic lesions were detected, in agreement with the majority of other animal models for RVFV, in which the virus was administered intranasally^29^. This potentially reflects the route of infection, where deposition in the nasal cavity in close proximity to the brain and subsequent rapid local replication influence the course of the disease, and the level of viremia could be insufficient to result in hepatic infection. It is also possible that other factors could play a role, however, as historically, multifocal hepatic necrosis and periportal mononuclear infiltrates have been described in ferrets following intranasal inoculation of pharyngeal washes from RVFV infected humans in one article from 1935^37^. Although not seen in our study, periportal lymphocytic infiltrates not attributed to RVFV RNA were reported in a recent experimental RVFV model in ferrets (Barbeau at al. 2020). Such observations have been described in ferrets challenged with SARS CoV and SARS CoV-2, although these can be a non-specific background finding in the laboratory ferret associated with other underlying viral infection^38–41^. Viral cellular attachment factor LRP1 plays a key role in RVFV entry into cells during the very early stages of RVFV infection^42^. Mice lacking LRP1 expression in hepatocytes have shown minimal RVFV replication in the liver, a longer time to death, and a tendency toward RVFV-induced neurological disease, rather than hepatic disease^42^, suggesting that LRP1 is essential for RVF hepatic disease in mice. Given the importance of LRP1 as a receptor for RVFV^20^, we examined the expression of LRP1 in the liver and brain. Strong LRP1 labelling was detected, but no reduction in labelling in the liver and the brain was detected between control tissues and those infected at different timepoints. However, the LRP1 gene transcriptional response clearly demonstrated a statistically significant reduction in LRP1 expression in CNS tissues by clinical endpoints. In comparison, there was no significant change in LRP1 transcript fold-change in the liver throughout the course of the study. These data highlight the tendency for neurological disease in the ferret model, rather than hepatic disease, and although the reason for LRP1 transcript downregulation in the CNS tissues tested is unclear, it may potentially constitute a host antiviral response that is initiated to restrict virus attachment to host cells in the CNS, to limit further viral dissemination. It is evident that the role of LRP1 in the host response to RVFV infection is complex, and it is likely to influence both inflammation and its suppression^43^. However, due to the ubiquitous LRP1 antibody detection throughout the tissues assessed, this is an area that requires further investigation.

The mechanism that initiates the apparent down-regulation of LRP1 in CNS tissues is also unclear, although it may potentially be induced by the pro-inflammatory cytokine response initiated by RVFV infection in CNS tissues, since pro-inflammatory cytokines, including IL-6 and TNF-α, have previously been shown to significantly down-regulate LRP1 in human primary microvascular endothelial cells (MVEC)^44^. In the current study, the detection of RVFV in the brain and spinal cord was accompanied by strong transcriptional upregulation for several innate immune response genes, including pro-inflammatory cytokines (TNF-α and IL-6), and the chemokine CCL5. Chemokine responses are essential for leukocyte recruitment to the sites of infection, and the release of cytokines primes the adaptive immune response^25^. However, pro-inflammatory cytokines released within the CNS may also contribute to disease pathology, rather than eliciting a protective effect, and the chemokine CCL5 produced predominantly by T-lymphocytes and monocytes may further recruit other leukocytes to the CNS to amplify the pro-inflammatory response^45^. Significant up-regulation of CCL5 transcripts was observed in CNS tissues at 6 − 8 DPI when compared with uninfected control animals and subclinical infected animals at 3 DPI, reflecting the severe inflammatory response in the CNS at clinical endpoints. There was no significant up-regulation of these genes at the earlier subclinical timepoint, suggesting that initiation of the innate immune response is at a low level during the subclinical phase of infection, before contributing to clinical and pathological changes and amplifying significantly towards clinical endpoint. Previous in vitro studies in infected human astrocytes have shown that despite RVFV inducing a strong type I interferon (IFN) and inflammatory response with production of the related messenger RNA (mRNA), modulation of mRNA transcription occurs (potentially through the NSs virulence factor) through inhibition of mRNA translocation from the nucleus to the cytoplasm, thus preventing establishment of the innate immune response and enabling efficient virus replication^35^. Hence, the host response is delayed, and viral replication and dissemination can proceed un-restricted, leading to significant tissue damage and eventually, profound dysregulated inflammatory responses and severe inflammation. However, in the current study, the immune transcript response was assessed only in CNS tissues and does not reflect the systemic cytokine profile. Intranasal inoculation of RVFV in ferrets also induced a neutralising antibody response in 1/3 infected ferrets at the pre-clinical time-point of 3 DPI, and all remaining infected ferrets by clinical endpoint. In human RVFV infections, a detectable neutralising antibody response is generally mounted within 4–6 days post-infection, sufficient to resolve clinical symptoms in many cases, although severe cases of RVF can occur despite an effective antibody response^23^. The latter scenario was observed in the ferret model, where a neutralising anti-RVFV antibody response was mounted, but this, along with the activation of an innate immune response, was unable to limit the spread of RVFV infection and dissemination into the CNS, with ultimately fatal consequences.

While it is acknowledged that studies of other viral infections in humans^46^ and in other species^47,48^, indicate gender influences in immune responses, prepubertal female ferrets were used in our model to avoid aggression and maintain wellbeing between subjects whilst infected with a high-consequence pathogen. Although comparison with previous ferret work may be more complex, our findings provide a gender-based background for future work.

In conclusion, intranasal inoculation of a high dose of RVFV ZH501 in ferrets resulted in rapid infection which was predominantly limited to the CNS and the eye. Our study suggests that RVFV invaded the CNS through retrograde, then anterograde dissemination within the brain and spinal cord, with minimal evidence of haematogenous dissemination. Although an innate immune response was mounted to infection and virus neutralising antibodies were produced, it was insufficient to halt infection. The ferret model recapitulates the encephalitic and ophthalmic manifestations of RVFV human infection, which is important for future validation of animal models of human disease for therapeutic interventions.

## Materials and methods

### Animals

Outbred female ferrets, aged approximately 18 weeks at the start of the study, were sourced from Marshall Farms Group Ltd, New York, USA. Ferrets were randomly assigned to groups of three and housed with food and water *ad libitum*. For temperature monitoring over the course of the study, microchips were implanted sub-dermally between the shoulder blades of each ferret. All methods were performed in accordance with relevant guidelines and regulations, with this study conducted under UK Home Office Licence PP2261426, and the experimental protocol approved by the Animal Welfare Ethical Review Body (AWERB) at the Animal and Plant Health Agency (APHA). A clinical signs monitoring schedule was implemented to detect early evidence of infection (See Supplementary Table S1) and humane clinical endpoints were defined and applied throughout experimental procedures.

### Virus isolate

Ferrets were inoculated with RVFV strain ZH501, originally derived from a fatal human case of haemorrhagic fever in Egypt in 1977 and isolated through mouse brain passage^49^. Working virus stocks were further passaged and titrated in Vero cells using standard techniques^50^.

### Experimental schedule

Following ferret acclimatisation, pre-challenge bleeds and swabs (nasal, oral, and rectal) were taken from all ferrets at −1-day post-inoculation (DPI) to establish baseline values. At 0 DPI, ferrets (*n* = 15) were inoculated intranasally with 10^7^ PFU RVFV strain ZH501 (titre previously confirmed through titration in Vero cells) in a total volume of 1 ml, with 0.5 ml administered per nostril, using a 1 ml pipette and sterile tips. Inoculation was performed under anaesthesia (isoflurane 4.5% induction, followed by medetomidine hydrochloride 1 mg/ml, butorphanol tartrate 10 mg/ml subcutaneously). The virus dose used was selected to^1^ mimic field inhalational exposure, since infected animals can be highly viraemic (10^6^−10^8^ PFU/mL)^51^, and^2^ to ensure the development of clinical disease (including neurological pathology) following intranasal inoculation, based on the findings of previously-published studies in ferrets^19^ and non-human primates (marmosets)^52^. One group of uninfected control ferrets (*n* = 3) were mock inoculated with 1 ml Eagle’s Minimum Essential Medium (E-MEM) (0.5 ml per nostril) and assessed in parallel. Ferrets were housed in groups of three, with infected and uninfected ferrets housed in two separate rooms. Cardboard boxes, white roll/tissue, tunnels, ferret balls, KONG toys and hammocks, and vitamin paste (Beaphar Uk Ltd, Suffolk UK) were used for enrichment.

Throughout the duration of the experiment, clinical signs, temperature, and bodyweight were recorded daily for all ferrets; swabs (oral, nasal and rectal) and blood samples were taken pre-challenge, and at terminal endpoint. At 3 DPI, one group of infected ferrets (*n* = 3) were humanely killed and subject to necropsy. All remaining infected ferrets were monitored daily and euthanised once clinical endpoints were reached (Supplementary Table S3) by intracardiac injection of an anaesthetic overdose (pentobarbitone sodium 20%) while under anaesthesia as described for intranasal inoculation. At the end of the experiment uninfected control ferrets (*n* = 3) were humanely killed and subject to necropsy. A range of tissues and swabs were taken from all infected and control groups.

### Pathology assessment of ferret tissues

At necropsy, a comprehensive list of tissue samples was collected into 10% neutral buffered formalin and fixed for at least five days. This included brain (olfactory bulb, cortex, cerebellum, brainstem), spinal cord (cervical, thoracic, and lumbar), eye, turbinates, tonsil, submandibular and retropharyngeal lymph nodes, thymus, tongue, trachea, lungs, heart, liver, spleen, kidney, pancreas, stomach, small intestine, mesenteric lymph node, colon, and urinary bladder. In CNS tissues, serial coronal sections were taken from the level of the olfactory bulb, frontal cortex including the rostral basal nuclei, caudal thalamus, occipital and temporal cortex, midbrain, cerebellum, and medulla oblongata. Transverse sections were taken of the proximal spinal cord (rostral to C1), and the cervical, thoracic, and lumbar spinal cord. CNS and other tissues were routinely processed and embedded in paraffin wax (Fisher Scientific, Loughborough, UK).

Serial sections were stained with haematoxylin and eosin (H&E) for histopathologic analysis and RVFV nucleoprotein or LRP1 antigen immunohistochemistry (IHC) from control and infected animals. For RVFV IHC, tissue sections of 4 μm thickness were dewaxed through xylene then rehydrated through absolute alcohol, quenched for endogenous peroxidase using 3% hydrogen peroxide in methanol (VWR International, Lutterworth, UK) for 15 min (min) at room temperature (RT), followed by epitope unmasking using a pH 9 buffer (VWR International, Lutterworth, UK) at 100 °C in a microwave for 10 min. Slides were blocked with normal goat serum and normal swine serum (NGt @ 1/66 and NSw @ 1/33; Vector Laboratories, Newark, USA) for 20 min at RT before assemblage into Shandon cover plates for IHC using the Sequenza system (Thermo Fisher Scientific, Runcorn, UK). Samples were then incubated with 1:12000 rabbit polyclonal anti-RVFV nucleoprotein antiserum^53^ (supplied by Australian Centre for Disease Preparation, Commonwealth Scientific Industrial Research Organisation, Australia) or with a matching isotype control (1:12000 rabbit IgG, Vector Laboratories, Newark, USA) on serial tissue sections, for 1 h at RT. This was followed by incubation with rabbit-specific Dako Envision+™ HRP-labelled polymer (Agilent Technologies, Cheadle, UK) with added normal goat and swine serum (NGt @ 1/66 and NSw @ 1/33; Vector Laboratories, Newark, USA) for 30 min at RT and visualised using 3,3-diaminobenzidine tetrahydrochloride (Sigma Aldrich, Gillingham, UK) for 10 min at RT. Tris-buffered saline (0.85% NaCl) with Tween (Fisher Scientific, Loughborough/VWR International, Lutterworth, UK) was used for rinsing sections between incubations and to dilute the primary antibody and block. Sections were then counterstained with Mayer’s haematoxylin (Surgipath, Leica Biosystems, Sheffield, UK), dehydrated and cleared in absolute alcohol and xylene, and glass coverslips mounted using dibutyl phthalate xylene (TCS Biosciences, Buckingham, UK). Positive control (infected mouse tissues [liver, spleen] and infected Vero cell pellets) and negative control (non-infected mouse tissues and isotypes) tissues were evaluated to confirm specificity of immunolabelling.

For LRP1 IHC, the primary antibody was optimised for IHC on the Bond RX autostainer (Leica Biosystems, Sheffield, UK). Sections were baked then dewaxed with Bond dewax solution (AR9222, Leica Biosystems, Sheffield, UK) before heat-induced epitope retrieval using Bond Epitope Retrieval Solution 1 (AR9961, Leica Biosystems, Sheffield, UK) for 20 min, and then incubated with a rabbit anti-human LRP1 primary antibody (Ab92544, Abcam, Cambridge, UK) at concentration 0.0512 ug/ml for 1 h or with concentrated matched isotype controls (Rabbit IgG; Vector Laboratories, Newark, USA). Labelling was amplified and demonstrated using the Bond Polymer Refine Detection kit (DS9800, Leica Biosystems, Sheffield, UK). The post-primary reagent was supplemented with normal ovine serum (1/33 (v/v) dilution; (Sigma-Aldrich, Gillingham, UK) and normal swine serum (1/33 (v/v) dilution; (Vector Laboratories, Newark, USA), and labelling was visualised using DAB chromogen and counterstained with Mayer’s haematoxylin. Bond Wash Solution (AR9590, Leica Biosystems, Sheffield, UK) was used for rinsing sections between incubations. Sections were then dehydrated, cleared and glass coverslips mounted using ClearVue mountant XYL (Epredia, Runcorn, UK).

To assess the association between pathology and detection of RVFV antigen, serial sections of H&E and IHC specimens were assessed by veterinary pathologists to correlate pathology and detection of RVFV antigen. Semi-quantitative scoring was performed on the histopathology and IHC results, where both inflammation and viral antigen labelling respectively were categorised as 0 (none), 1 (sparse), 2 (mild), 3 (moderate), and 4 (abundant).

### Molecular detection of RVFV in ferret tissues and swabs

RNA was extracted from tissues and swabs using TRIzol™ or TRIzol™ LS (Invitrogen) according to manufacturers’ instructions. Eluted RNA was assessed using a RVFV and β-actin TaqMan RT-PCRs, with the iTaq™ Universal Probes One-Step Kit (Bio-Rad) and previously published primers that amplify a 94-base pair (bp) fragment of the RVFV M segment encoding the glycoprotein GP2^54^. The multi-species β-actin primers and probe were as detailed in^55^. Briefly, a RVFV master mix was prepared; per reaction 1000 ng RNA was combined with 10 µl of 2x iTaq Universal Probes Reaction Mix (Bio-Rad), 0.5 µl of iScript Reverse Transcriptase (Bio-Rad), 18 pmol each of the forward and reverse primers (RVS_m and RVAs_m respectively), 5 pmol of probe RVP_m, made up to a total of 20 µl with nuclease-free water. Alongside this, a β-actin master mix was prepared; per reaction 1000 ng RNA was combined with 10 µl of 2x iTaq Universal Probes Reaction Mix (Bio-Rad), 0.5 µl of iScript Reverse Transcriptase (Bio-Rad), 10 pmol each of primers BatRat Beta Actin Int, BatRat Beta Actin Rev, 2.5 pmol of BatRat Beta Actin Probe, and made up to a total of 20 µl with nuclease-free water. Cycling conditions were 50 °C for 10 min (reverse transcription), 95 °C for 5 min, 45 cycles of 95 °C for 15 s and 55 °C for 30 s, using the FAM and ROX filters for RVFV and β-actin, respectively. The normalisation of the RVFV Ct values to β-actin was determined by calculating the 75th percentile of the β-actin Ct values, then dividing the RVFV Ct values by the resulting β-actin 75th percentile value. For graphical representation, results are displayed as reciprocal normalised Ct values, i.e., 1/normalised Ct.

### Molecular detection of innate immune transcripts in tissues

RNA transcript modulation for immune markers was assessed using previously described methods^56^, based on SYBR Green assays to determine mean transcript fold changes, normalised against β-actin, and in relation to the uninfected control group. Transcript-specific primer pairs were designed for chemokine ligand 5 (CCL-5), interleukin 6 (IL-6), tumour necrosis factor-alpha (TNF-α) and LRP1, and are detailed in Supplementary Table S2. These innate immune transcripts were selected as they have been previously shown to be representative markers of viral encephalitis in a range of mammalian hosts with several neurotropic viruses^57–60^, and providing evidence for activation of the innate immune response.

### Serological analysis

Serum samples taken at terminal endpoint were assessed for viraemia by plaque assay on Vero C1008 cells using standard techniques to determine virus titre as plaque forming units per ml (PFU/ml)^50^. Briefly, 10-fold serum dilutions (11 dilutions, up to 10^− 11^) were prepared in Eagle’s Minimum Essential Medium (Gibco [Thermo Fisher Scientific], Paisley, UK) without serum (E-MEM-WOS), containing 100 U/ml penicillin, 100 µg/ml streptomycin and 25 U/ml nystatin, and incubated for 60 min (37 °C, 5% CO_2_) with 1-day old monolayers of Vero C1008 cells in 12-well cell culture plates (3 × 10^5^ cells/ml, in a 3 ml volume per well). Following addition of an overlay containing 7.5 g carboxymethylcellulose (CMC) (Sigma Aldrich, Merck KGaA, Poole, UK), 1X Minimum Essential Medium Eagle with Earle’s salts (Sigma Aldrich, Merck KGaA, Poole, UK), 1% foetal bovine serum (Sigma Aldrich, Merck KGaA, Poole, UK), 0.2% sodium bicarbonate (Sigma Aldrich, Merck KGaA, Poole, UK), 0.02 M HEPES ((4-(2-hydroxyethyl)−1-piperazineethanesulfonic acid) (Sigma Aldrich, Merck KGaA, Poole, UK), 2 mM L-glutamine (Sigma Aldrich, Merck KGaA, Poole, UK), and penicillin/streptomycin (Sigma Aldrich, Merck KGaA, Poole, UK), plates were further incubated for 6 days. Cell monolayers were fixed with 10% neutral buffered formalin, and stained with 2.3% crystal violet solution (Sigma Aldrich, Merck KGaA, Poole, UK) to enable plaque visualisation and counting. Titres were determined as plaque forming units per mL (PFU/mL). RVFV neutralising antibodies were measured by plaque reduction neutralisation test (PRNT) as previously described^61^, using Vero C1008 cells and RVFV ZH501. Briefly, 2-fold serum dilutions (eight dilutions up to 1/1280) were prepared in E-MEM-WOS in a 96-well microtitre plate, and incubated with RVFV ZH501 (diluted to a concentration that gives 50–70 plaques per well, determined by plaque assay as detailed above) for 60 min (37 °C, 5% CO_2_). For each sample to test, the serum dilutions/virus combination were then transferred to eight wells of 12-well plates with 1-day old cultures of Vero C1008 cells; triplicate virus control wells were included in each plate, along with a media-only uninfected control well, before incubation for a further 60 min (37 °C, 5% CO_2_). Plates were then processed as detailed above; addition of CMC overlay and incubation for 6 days, followed by fixation and staining of the cell monolayers and plaque counting. PRNT_90_ titre was determined as the dilution of a sample at which a 90% reduction in plaques occurs, in comparison to mean number of plaques counted in triplicate virus control wells on each plate.

### Statistical analysis

All statistical analyses were undertaken using GraphPad Prism 8.4.2. The unpaired t-test (two-tailed distribution) was used to assess statistically significant differences between mean body temperature for uninfected and RVFV-infected ferrets at each timepoint, and the statistical significance between preclinical and clinical detections of viral RNA by RT-PCR in swabs and tissues. Statistically significant differences in percentage weight change (in relation to baseline values at 0 DPI) for uninfected ferrets and RVFV-infected ferrets at each timepoint post-infection (mean ± Standard Error of the Mean [SEM]) were also assessed using the unpaired t-test (two-tailed distribution). The unpaired t test was considered appropriate for these analyses, as two independent groups were assessed at each timepoint, where both populations were assumed to have similar standard deviations. For immune transcripts, statistical comparisons of mean fold change (in comparison to the uninfected control group) were assessed using a one-way ANOVA for multiple comparisons (three independent groups) with Tukey’s comparison of means, hence controlling for Type I errors. The level of significance was set at *p*-value < 0.05. Calculations of Means and SEM were undertaken in Microsoft Excel.

## Data Availability

The data that support the findings of this study are available from the corresponding author, upon reasonable request.

## ementary Information

Below is the link to the electronic supplementary material.

Supplementary Material 1

## Abbreviations

APHA: Animal and Plant Health Agency
AWERB: Animal Welfare Ethical Review Body
β-actin: Beta actin
BBB: Blood–brain barrier
bp: Base pairs
CCL5: Chemokine ligand 5
CNS: Central nervous system
CSF: Cerebrospinal fluid
DPI: Days post-infection
E-MEM: Eagle’s minimum essential medium
H&E: Haematoxylin and eosin
HPAI: Highly pathogenic avian influenza
IFN: Interferon
IHC: Immunohistochemistry
IL-6: Interleukin 6
LRP1: Low-density lipoprotein receptor-related protein 1
mRNA: Messenger ribonucleic acid
MVEC: Microvascular endothelial cells
NSs: Non-structural protein on the S segment
OEC: Olfactory ensheathing cells
PFU: Plaque forming units
PM: Postmortem
PRNT: Plaque reduction neutralisation test
RNA: Ribonucleic acid
RT: Room temperature
RT-PCR: Reverse transcription polymerase chain reaction
RVFV: Rift Valley fever virus
SARS CoV: Severe acute respiratory syndrome coronavirus
SEM: Standard error of the mean
TNF-α: Tumour necrosis factor-alpha
