# Supplementary material for: Intranasal infection of Rift Valley fever virus in the ferret model suggests multiple routes of neuroinvasion with consequential encephalitis and ophthalmitis
Source: Sci Rep. 2026 May 27;16:27071. doi: 10.1038/s41598-026-53431-5 (PMC13522356; doi:10.1038/s41598-026-53431-5)
Supplement: Supplementary file 1 [file 41598_2026_53431_MOESM1_ESM.docx]

**Supplementary material**

**Supplementary Table S1.** Clinical scoring criteria for RVFV ferret study.

| **Parameter** | | **Criteria** | **Score** |
| --- | --- | --- | --- |
| **1** | **Alertness** | Attentive (curious, alert, active) | 0 |
|  |  | Slightly reduced activity, hesitant, disinterested | 1 |
|  |  | Significantly reduced activity | 2 |
|  |  | Inactive, recumbent, even when stimulated | 3 |
| **2** | **Weight loss** | No weight loss compared to day 0 | 0 |
|  |  | Weight loss <5% compared to day 0 | 1 |
|  |  | Weight loss 10% compared to day 0 | 2 |
|  |  | Weight loss >15% compared to day 0 | 3 |
| **3** | **Body tension** | Relaxed | 0 |
|  |  | Slightly hunched back | 1 |
|  |  | Flaccid or hunched / rigid body | 3 |
| **4** | **Breathing** | Normal | 0 |
|  |  | Sneezing / coughing | 1 |
|  |  | Raised respiration | 2 |
|  |  | Breathing difficulties, wheezing | 3 |
| **5** | **Coat / skin** | Smooth, flat coat | 0 |
|  |  | Slightly roughened coat, piloerection, lack of grooming, reddening at injection site | 1 |
|  |  | Rough, dull looking coat | 2 |
| **6** | **Eyes** | Eyes clear and bright | 0 |
|  |  | Clear discharge from eyes or nose, squinting | 1 |
|  |  | Conjunctivitis, yellow discharge from eyes and nose | 2 |
| **7** | **Appetite / drinking** | Normal, eats most food offered | 0 |
|  |  | Little food eaten, moderate interest in treats when offered | 1 |
|  |  | Shows no interest in food or treats | 2 |
|  |  | No food or water consumed in 24hrs | 3 |
| **8** | **Gastrointestinal** | Normal faecal consistency | 0 |
|  |  | Vomiting or soft stool | 1 |
|  |  | Runny stool, bloody stool | 2 |
| **9** | **Temperature** | <1°C (+/-) change from baseline | 0 |
|  |  | + 1-1.5°C change from baseline | 1 |
|  |  | More than +1.5°C change from baseline | 2 |
|  |  | >41.0°C or decrease of 1°C below baseline | 3 |
| **10** | **Neurological** | Normal behaviour, carriage of body and limbs, and locomotion | 0 |
|  |  | Tremors | 1 |
|  |  | Limb weakness but moving around cage with slight head tilt (able to feed and drink) | 2 |
|  |  | Seizures, ataxia, severe head tilt | 3 |

**Supplementary Table S2.** Primer sequences for innate immune host transcript analysis of ferret tissue.

| **Transcript** | **Primer name** | **Primer sequence (5’-3’)** | **Melting temperature (°C)** | **Amplicon size (bp)** |
| --- | --- | --- | --- | --- |
| **CCL-5** | FRT_CCL5 For | CTGCTTTGCCTACATCTC | 55.9 | 146 |
|  | FRT_CCL5 Rev | GCACCCATTTCTTCTGTG | 59.2 |  |
| **IL-6** | FRT_IL6 For | GAAGACTTCATTAGGTTCATCC | 58.2 | 201 |
|  | FRT_IL6 Rev | CTGAAGACCGGTAGTGATTC | 58.8 |  |
| **LRP-1** | FRT_Lrp1 For | GTAACCTGCAGTGCTTCAAT | 59.4 | 121 |
|  | FRT_Lrp1 Rev | CAGTACTCCCAGCACTGG | 59.4 |  |
| **TNF-α** | FRT_TNFalpha For | AGCCACCACACTGTTCTG | 59.9 | 254 |
|  | FRT_TNFalpha Rev | GCACTATTAGCTGGTTGTCTG | 59.0 |  |

Abbreviations: bp, base pairs.

**Supplementary Table S3.** Ferret clinical signs, clinical scores, and gross findings.

| **Ferret No** | **DPI** | **Clinical comments** | **Clinical Score at euthanasia** | **PM Changes** |
| --- | --- | --- | --- | --- |
| **1** | 3 | Pyrexia day two | 0 | Mild meningeal hyperaemia |
| **2** | 3 | No clinical signs | 0 | Mild meningeal hyperaemia |
| **3** | 3 | No clinical signs | 0 | Mild meningeal hyperaemia |
| **4** | 6 | Shivering, vomiting, hypersalivation, random bursts of activity, rear in air holding R paw up, squinting, tremors, irregular breathing, unable to stand for long. | 10 | Severe meningeal hyperaemia |
| **5** | 7 | Pyrexia d6, persistent. Unusual behaviour, rapid breathing, squinting | 5 | Moderate meningeal hyperaemia |
| **6** | 7 | Pyrexia d6, unusual behaviour | 3 | Moderate meningeal hyperaemia |
| **7** | 7 | Pyrexia d5, persistent, unable to walk, tense body | 11 | Severe meningeal hyperaemia |
| **8** | 7 | Pyrexia d6, lethargic, less responsive | 8 | Mild meningeal hyperaemia |
| **9** | 7 | Pyrexia d5, persistent. Squinting, unsteady gait | 14 | Moderate meningeal hyperaemia |
| **10** | 7 | Pyrexia d5, persistent. Seizures, hypersalivation, vomiting, lifting front paw, reclining | 11 | Severe meningeal hyperaemia |
| **11** | 7 | Limb weakness, progressing to inability to move. | 10 | Moderate meningeal hyperaemia |
| **12** | 7 | Pyrexia d6, limb weakness, confusion | 10 | Moderate meningeal hyperaemia |
| **13** | 7 | Pyrexia d6, laboured breathing, incoordination, poor balance | 15 | Moderate meningeal hyperaemia |
| **14** | 8 | Pyrexia d6 persistent. Squinting, overgrooming | 10 | Mild meningeal hyperaemia |
| **15** | 8 | Pyrexia d5, intermittent, incoordination, circling | 9 | Moderate meningeal hyperaemia |
| **16** | 3 | No clinical signs | 0 | No lesion detected |
| **17** | 3 | No clinical signs |  |  |
| **18** | 3 | No clinical signs |  |  |

Abbreviations: DPI, days post-infection; PM, postmortem.

**Supplementary Table S4**. Detection of RVFV in swabs by RT-PCR, from intranasally infected ferrets.

| **Swab** | **RVFV 3 DPI**  **(*n*=3)** | **RVFV 6−8 DPI**  **(*n*=12)** |
| --- | --- | --- |
| Oral | 3 (100) | 5 (41.7) |
| Nasal | 3 (100) | 11 (91.7) |
| Rectal | 0 (0) | 3 (25) |

Number of positive ferrets per swab type shown, with percentages shown in parentheses. Total number of ferrets sampled denoted by *n*. There was no detection of viral RNA in swabs taken from uninfected ferrets (*n*=3).

**Supplementary Table S5.** Shedding profile for individual ferrets following intranasal inoculation with RVFV.

|  |  |  | **Ct value** | | | | | |
| --- | --- | --- | --- | --- | --- | --- | --- | --- |
|  | **Endpoint (DPI)** | **Ferret ID** | **RVFV-specific RT-PCR** | | | ***Β*-actin RT-PCR** | | |
|  |  |  | **Oral swab** | **Nasal swab** | **Rectal swab** | **Oral swab** | **Nasal swab** | **Rectal swab** |
| **Uninfected** | 10 | F401 | *No Ct* | *No Ct* | *No Ct* | 36.19 | 43.29 | 31.45 |
|  |  | F026 | *No Ct* | *No Ct* | *No Ct* | 33.82 | 36.98 | 35.76 |
|  |  | F216 | *No Ct* | *No Ct* | *No Ct* | 36.88 | 34.72 | 39.61 |
| **RVFV-infected** | 3 | F004 | 30.22 | 36.59 | *No Ct* | 35.74 | 35.04 | 38.07 |
|  |  | F456 | 28.83 | 33.15 | *No Ct* | 33.77 | 32.16 | 41.49 |
|  |  | F973 | 28.39 | 35.77 | *No Ct* | 33.98 | 36.03 | 36.30 |
|  | 6 | F202 | 29.88 | 21.70 | 39.21 | 33.47 | 35.85 | 39.31 |
|  | 7 | F025 | 41.24 | 28.57 | *No Ct* | 36.62 | 32.12 | 42.89 |
|  |  | F359 | 36.01 | 28.38 | *No Ct* | 33.87 | 32.39 | 39.87 |
|  |  | F706 | 39.96 | 27.51 | *No Ct* | 35.95 | 34.05 | 41.59 |
|  |  | F074 | *No Ct* | 31.25 | *No Ct* | 35.42 | 35.13 | 34.31 |
|  |  | F365 | *No Ct* | 34.52 | *No Ct* | 33.63 | 35.57 | 35.38 |
|  |  | F785 | 32.72 | 34.11 | 36.36 | 35.14 | 35.64 | 35.60 |
|  |  | F689 | *No Ct* | 39.60 | *No Ct* | 34.51 | 37.62 | 36.97 |
|  |  | F118 | *No Ct* | 33.20 | *No Ct* | 34.34 | 35.48 | 37.51 |
|  |  | F448 | *No Ct* | 34.81 | *No Ct* | 37.64 | 35.98 | 37.72 |
|  | 8 | F952 | *No Ct* | 35.58 | *No Ct* | 37.94 | 34.40 | 37.50 |
|  |  | F008 | *No Ct* | *No Ct* | 37.84 | 34.81 | 35.88 | 36.49 |

Ct values determined through assessment of post-mortem swabs at clinical endpoints (DPI) by RVFV-specific RT-PCR. Abbreviations: DPI, days post-infection.

**Supplementary Table S6.** Evidence of neutralising antibody response following intranasal inoculation with RVFV.

|  | **Endpoint (DPI)** | **Ferret ID** | **PRNT_90_ titre (PFU/ml)** | |
| --- | --- | --- | --- | --- |
|  |  |  | **Pre-bleed** | **Post-mortem bleed** |
| **Uninfected** | 10 | F401 | *Negative* | *Negative* |
|  |  | F026 | *Negative* | *Negative* |
|  |  | F216 | *Negative* | *Negative* |
| **RVFV-infected** | 3 | F004 | *Negative* | 1:20 |
|  |  | F456 | *Negative* | *Negative* |
|  |  | F973 | *Negative* | *Negative* |
|  | 6 | F202 | *Negative* | 1:40 |
|  | 7 | F025 | *Negative* | 1:40 |
|  |  | F359 | *Negative* | 1:40 |
|  |  | F706 | *Negative* | 1:40 |
|  |  | F074 | *Negative* | 1:40 |
|  |  | F365 | *Negative* | 1:80 |
|  |  | F785 | *Negative* | 1:40 |
|  |  | F689 | *Negative* | 1:40 |
|  |  | F118 | *Negative* | 1:40 |
|  |  | F448 | *Negative* | 1:40 |
|  | 8 | F952 | *Negative* | 1:10 |
|  |  | F008 | *Negative* | 1:80 |

Neutralising antibody titre determined through PRNT_90_ (PFU/ml) of pre-bleeds (taken at 0 DPI) and post-mortem bleeds at clinical endpoint. Abbreviations: DPI, days post-infection.

**Supplementary figures**

**1cm**

**1cm**

**Supplementary Fig. S1.** Gross image of the ferret brain at 6 DPI. Mild to moderate diffusely reddened meninges in all infected ferrets **(A)**, compared to uninfected control brain **(B)**.

**Supplementary Fig. S2 -** Detection of viral RNA (reciprocal Ct normalised against β-actin) in oral swabs and nasal swabs. Molecular detection at 3 DPI (subclinical [*n*=3], green bars) and at 6−8 DPI (clinical endpoint [*n*=12], orange bars), providing evidence of a reduction in virus shedding through the oral route during the clinical phase of infection (6−8 DPI). Statistical differences between subclinical and clinical timepoints were determined by unpaired t-test (two-tailed distribution). * *p*<0.05.

**Supplementary Fig. S3.** Schematic of the pathogenesis of neuroinvasion following intranasal inoculation of ferrets with RVFV, highlighting the multiple routes of virus entry into the CNS. *Created with BioRender.com* . Solid arrows indicate primary pathways both evidenced and proposed by this study, and dotted arrows suggest other potential secondary pathways. Blue arrows indicate retrograde neurological pathways, and green arrows represent anterograde neurological pathways. Red arrows indicate haematogenous pathways. Abbreviations: OECs - olfactory ensheathing cells. CN – cranial nerve.
